# Two Cases of Grave Complications of Purulent Ear Disease

**Published:** 1906-06-30

**Authors:** J. Stoddart Barr

**Affiliations:** Assistant Surgeon at the Glasgow Ear, Nose, and Throat Hospital.


### TWO CASES OF GRAVE COMPLICATIONS OF PURULENT EAR DISEASE.

By J. Stoddart Barr, M.B., Ch.B., Assistant Surgeon at the Glasgow Ear, Nose, and Throat Hospital.

The two following cases are illustrations of important symptoms in connection with the complications of middle ear disease. The first case had repeated rigors, which continued for as long a period as eleven days. The second case illustrates the occasional and puzzling presence of optic neuritis in association with disease of the middle ear in which there is no evidence that inflammation has spread to the meninges or brain.

### I. Septic Thrombosis of the Lateral Sinus, secondary to Otitis Media Septic Infarctions of the Right Lung.

The patient was a little girl, seven years of age, who had had a continuous discharge from the left ear for a year. During the eleven days before admission she had a dozen severe rigors, with violent oscillations of temperature, from normal to 104° F. There was also frontal headache and pain in the left side of the neck. She vomited once or twice at the beginning of the illness. The discharge from the ear was profuse and fœtid. There was evidence of the early stage of optic neuritis in both eyes. The breathing was accelerated with frequent cough. A few moist râles were heard and also tubular breathing over a restricted area around the inferior angle of the right scapula. The radical mastoid operation was performed, clearing out pus and granulation tissue. The sigmoid sinus was exposed and found converted into a yellowish sloughy-looking mass surrounded by and partly filled with pus. The bulb of the internal jugular contained solid thrombus. The sinus was slit open backwards and cleared of septic disintegrated thrombi to within $1\frac{1}{2}$ inches of the torcular, when a gush of blood appeared. After the operation the pulmonary symptoms continued to increase, and the patient died two and a half days after admission to the hospital. Post-mortem examination was conducted by Dr. James Walker, and showed well-marked septic infarctions of the right lung. Bacteriological examination showed the presence of pneumococci and streptococci and also a large bacillus, which did not grow on the culture media, and did not give the staining reactions of the acid-fast group of organisms.

In this case it is highly probable that had operative treatment been employed a few days earlier, before the pulmonary infection had supervened, a different result might reasonably have been expected. It should be emphasised that, when rigors with violent oscillations of temperature appear in connection with a purulent middle ear disease operative treatment is urgently called for. Judging from this case and others of a similar kind, the grave significance of this symptom in these ear cases is not yet duly recognised by some practitioners. Operation carried out early is probably more successful than in any other form of intracranial complications due to ear disease; hence the vital importance of early recognition of the condition present.

### II. A Case of Otitis Media Extra-dural Abscess associated with Paralysis of the Sixth Cranial Nerve and Double Optic Neuritis. Operation and Recovery.

The patient was a lad of 17 years of age who suffered from purulent right middle ear disease for sixteen months before admission. He came to the hospital owing to headache, diplopia, paralysis of external rectus (sixth nerve), and double optic neuritis. The radical mastoid operation cleared out cholesteatomatous masses from the antrum, aditus, and the attic of the tympanum. The opening of the sigmoid groove gave exit to a collection of pus between the sigmoid part of the lateral sinus and the bone. The sinus wall was involved and covered with granulation tissue, but there being no signs of general septic infection, it was not opened. There had been no pain over nor behind the mastoid area, and the temperature was normal from the time of admission. After the operation the paralysis of the sixth nerve gradually, but slowly, passed off. The optic neuritis for a few weeks became more marked with occurrence of hæmorrhagic spots, and now, four months after the operation, the discs are undefined, with white spots and a tendency to atrophy, but the vision is so far unimpaired.

*Remarks.*—The pathological connection between the septic pachy-meningitis at the sinus and the lesions causing the ocular phenomena is not very clear. The most ready explanation is that a limited basal lepto-meningitis originated in the region of the sigmoid groove and extended and involved the sheath of the sixth nerve or the more distant optic commissure, either by pressure or by producing an infective neuritis. There is also the possibility of the formation of a thrombus in the cavernous sinus, originating in the sigmoid and by pressure involving the sixth nerve as it lies in the cavernous sinus, or by producing stasis of the blood from the eye, causing optic neuritis. It is also to be remembered that optic neuritis seems to occasionally occur in connection with simple purulent middle ear disease presenting no intracranial symptoms.

